# Identification of a Germline *XAF1* Mutation in Patients With Gastrointestinal Cancers

**DOI:** 10.1155/humu/4279712

**Published:** 2026-05-04

**Authors:** Guan-Xin Xu, Hang Zhang, Chang-Xing Wang, Ying-Zhi Zhang, Ping-Ping Lv, Chun Feng, Yao Ning, Miao Shen, Sai Zhang, Min Jin, Dan-Qing Yu

**Affiliations:** ^1^ Department of Thoracic Surgery, The Second Affiliated Hospital of Zhejiang University, Hangzhou, Zhejiang Province, China, z2hospital.com; ^2^ Department of Gastrointestinal Surgery, The Second Affiliated Hospital of Zhejiang University, Hangzhou, Zhejiang Province, China, z2hospital.com; ^3^ Department of Pathology, The Second Affiliated Hospital of Zhejiang University, Hangzhou, Zhejiang Province, China, z2hospital.com; ^4^ Department of Laboratory Medicine, The Second Affiliated Hospital of Zhejiang University, Hangzhou, Zhejiang Province, China, z2hospital.com; ^5^ Department of Reproductive Medicine, Women’s Hospital, School of Medicine, Zhejiang University, Hangzhou, Zhejiang Province, China, zju.edu.cn; ^6^ Key Laboratory of Reproductive Genetics, Ministry of Education (Zhejiang University), Hangzhou, Zhejiang Province, China; ^7^ Department of Reproductive Medicine, The Second Affiliated Hospital of Zhejiang University, Hangzhou, China, z2hospital.com; ^8^ Department of Gynaecology and Obstetrics, The First People’s Hospital of Jiande, Hangzhou, Zhejiang Province, China, ncchd.go.jp

## Abstract

**Background:**

Gastrointestinal (GI) cancers can be attributed to the interplay between genetic and environmental factors. To date, apart from certain cancer syndromes, the genetic factors underlying familial GI cancers have not been clearly elucidated.

**Methods:**

Blood samples were collected from six members of a family with GI cancer for whole exome sequencing to identify suspicious germline mutations. Subsequently, 148 patients with GI cancers (including esophageal and gastric cancers) and 283 cancer‐free patients were recruited. The frequency of the suspected mutations in both groups was determined using Sanger sequencing. Furthermore, immunofluorescence (IF) assays for XAF1 protein expression were performed in paraffin‐embedded surgically resected tumor tissues from patients with GI cancer, with or without the mutation.

**Results:**

In a family with GI cancer, we identified a mutation of *XAF1* (c.454+1372G>A), which is a nonsense mutation in Exon 4b that results in a truncated XAF1 Isoform 5. Sanger sequencing of sporadic cancer patients and cancer‐free populations further verified that the frequency of this mutation was enriched in patients with GI cancer. Additionally, IF assays revealed that XAF1 protein expression was lower in the mutated group than in the nonmutated group.

**Conclusion:**

Our study provides evidence that a *XAF1* mutation (c.454+1372G>A) leads to repressed expression of XAF1 and is associated with a predisposition to GI tumorigenesis, especially in esophageal and gastric cancers.

## 1. Introduction

Gastrointestinal (GI) cancers encompass a spectrum of malignancies affecting the digestive system ([1]). While most GI cancers are sporadic, approximately 3%–10% of gastric, liver, or other GI cancers exhibit familial clustering [2, 3]. The genetic alterations involved in GI cancers are mainly observed in oncogenes, tumor suppressor genes, and DNA repair genes. Some common genes are involved in various GI cancers [1], including the *MDM2* T309G polymorphism, which has been linked to susceptibility to colon [4], gastric [5], and hepatocellular cancers [6]. Biallelic mutations (Muts) in *MUTYH* are associated with MUTYH‐associated polyposis, which increases the risk of colorectal and duodenal cancer [7, 8]. Other genes, including *OGG1*, *XRCC1*, *PARP1*, *TGFβ*, *TGFBR1*, and *SMAD7*, exhibit associations with multiple GI cancers [9–14]. Germline Muts in *TP53* are the causal genetic defects in Li–Fraumeni syndrome, which is associated with gastric cancer, esophageal cancer, colorectal cancer, and hepatocellular carcinoma [15–17]. The *TP53* gene is located on chromosome 17p13.2, and loss of heterozygosity (LOH) has been observed in many cancer cell lines, suggesting that this region is rich in tumor suppressors. *XAF1* also localizes to chromosome 17p13.2, which is telomeric to TP53, at a distance of 3cM. XAF1 has been credited as a tumor suppressor and was initially identified as an antagonist of XIAP. XAF1 interacts with p53, IFN, and MT2A, resulting in apoptosis. XAF1 is widely expressed in normal and fetal tissues but is expressed at lower levels in most human cancer cell lines [18, 19]. Alternative regulatory mechanisms of XAF1, such as epigenetic modifications and altered splicing variants, have been reported [20–24]. However, only a few perturbations have been observed in this gene [22, 25].

In our study, we evaluated a three‐generation pedigree that includes eight patients with GI cancer, in which we identified a Mut in *XAF1*. We also provide data on the frequency of this Mut in *XAF1* in a group of patients with unselected GI cancers and cancer‐free controls. Furthermore, we compared the expression of XAF1 protein in patients with or without *XAF1* Muts using immunofluorescence of surgically removed tumor tissues. In this study, we aimed to elucidate the role of the identified *XAF1* Mut in conferring genetic susceptibility to GI cancer.

## 2. Results

### 2.1. Participation of Family With High GI Cancer Incidence

**Figure 1:**
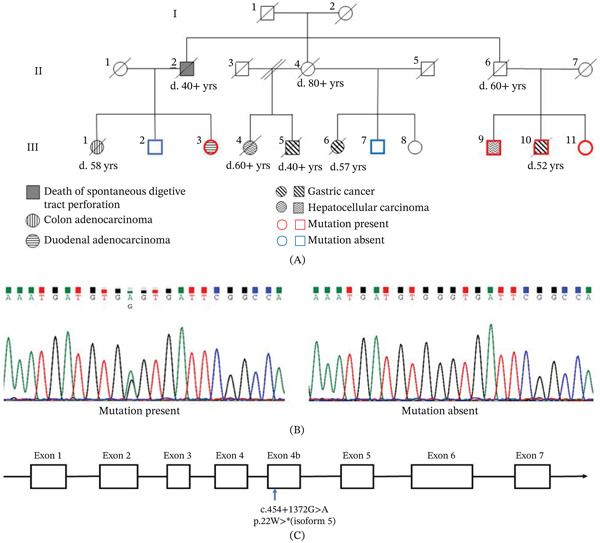
Pedigree of a Chinese family with high GI cancer frequency. (A) The pedigree chart. Different symbols denote various cancer types. Age at death is indicated below each symbol. Heterozygous mutations are represented by (+/−). Whole exome sequencing was performed on seven members: Five patients with a heterozygous mutation in *XAF1* are marked in red, and two individuals without the mutation are marked in blue. (B) The sequence of *XAF1* from the family members was validated using Sanger sequencing. (C) Schematic diagram of the *XAF1* mutation site.

The pedigree of a family in which early GI cancer diagnosis occurred frequently was obtained. In Generation III, 7 of the 11 members were diagnosed with GI cancers before 60 years of age, as shown in Figure 1A. All diagnoses were verified via pathological examination. At the time of recruitment, four members of Generation III had died because of GI cancers. Of the remaining seven members, III:8 refused to undergo peripheral blood collection and only reported a medical history without any cancer diagnosis. Blood samples were collected from six members, of whom one was diagnosed with colorectal cancer, one with hepatocellular cancer, one with gastric cancer, and three without any cancer diagnosis at the time of the report. However, it is difficult to trace the medical histories of Generations I and II. The medical histories of Generation II were based on the reports of their children. In particular, II:2 died in his 40s due to a spontaneous digestive tract perforation over 40 years prior to the initiation of this study. The clinical and genotypic characteristics of the patients are shown in Table 1.

**Table 1:** Clinical characteristics and genotype information of individuals in the GI pedigree.

Pedigree ID	Sex	*XAF1* genotype	Age at genotyping	Diagnosis	Age at diagnosis	Age of death	Cause of death
II:2	Male	NA	NA	NA	NA	40+	Acute intestinal perforation
II:4	Female	NA	NA	NA	NA	60+	NA
II:6	Male	NA	NA	NA	NA	60+	Acute myocardial infarction
III:1	Female	NA	NA	Colon adenocarcinoma	58	59	COAD
III:3	Female	+/−	61	Duodenal adenocarcinoma	57	NA	NA
III:4	Female	NA	NA	Hepatocellular carcinoma	60+	60+	HCC
III:5	Male	NA	NA	Gastric cancer	40+	40+	GC
III:6	Male	NA	NA	Gastric stromal cancer	56	58	GC
III:9	Male	+/−	53	Hepatocellular carcinoma	46	NA	NA
III:10	Male	+/−	51	Stomach adenocarcinoma	50	51	STAD
III:11	Female	+/−	49	NA	NA	NA	NA
III:2	Male	−/−	63	NA	NA	NA	NA
III:7	Male	−/−	62	NA	NA	NA	NA

*Note:* (+/−) indicates a heterozygous mutation, while (−/−) denotes the wild type.

Abbreviations: COAD, colon adenocarcinoma; GC, gastric carcinoma; HCC, hepatocellular carcinoma; IGC, intestinal gastric cancer; NA, not applicable; STAD, stomach adenocarcinoma.

### 2.2. Whole Exome Sequencing (WES) and Sanger Sequencing Validation

DNA from six members of Generation III was enriched and sequenced by WES, as described in the Methods section. Heterozygous variance in the *XAF1* gene (NM_001353137.2: c.454+1372G>A) was shared by all three participants with GI cancer and one without cancer (III:11), who was 50 years old at the time of sequencing. The other two individuals without cancer (III:2 and III:7) did not harbor this variant. Allele frequencies were 0.00006, 0.0009, and 0.000185 in the Exome Aggregation Consortium (ExAC), 1000 Genomes Project, and gnomAD databases, respectively. In the dbSNP database, the ID was rs117407731, which has been reported as a stop‐gain Mut.

XAF1 shows variable expression spectra of different isoforms, and shorter transcripts have higher expression in tumor samples [26, 27]. There are 10 transcripts of the *XAF1* gene, as shown in Table S1. The c. 454+1372G>A variant is located in Exon 4b and results in a premature termination codon for Transcript Variants 9 (NM_001353137.2) and 10 (NM_001353138.2). These two transcripts translate into XAF1 Isoform 5 (NP_001340066.1, NCBI Reference Sequence), whereas the variant c.454+1372G>A leads to a truncated Isoform 5 protein (p 22 W >∗) or nonsense‐mediated decay of mRNA. XAF1 protein expression in normal gastric and esophageal tissues is shown in Figure S1, demonstrating a relatively high abundance of Isoform 5. As previously reported, the full‐length protein contains seven zinc finger (ZF) domains with distinct boundaries: the highly compact N‐terminal domain (NTD) ZFs 1–5, middle domain (MD) ZF6, and C‐terminal domain (CTD) ZF7. The XAF1 Isoform 5 contains ZF6 and 7, among which ZF7 is identified as the domain responsible for the XAF1‐GRP78 interaction and thus mediates apoptosis [28].

From Generation III, three individuals had been diagnosed with gastric cancer. III:10 diagnosed with gastric adenocarcinoma after gastroscopy revealed a mass in the lesser curvature of the gastric antrum. A subsequent CT scan suggested multiple metastases to the perigastric and retroperitoneal lymph nodes, left adrenal gland, and omentum. The patient subsequently received six cycles of XELOX (capecitabine plus oxaliplatin) chemotherapy. However, a subsequent CT scan suggested tumor progression with increased metastasis and ascites. Unfortunately, the patient died 7 months after diagnosis at the age of 51 years. III:10 was the first to visit our hospital and report their family histories. WES of III:10 revealed a heterozygous variation. Sanger bidirectional sequencing confirmed that there was a heterozygous variant of c.454+1372G>A. III:5 was diagnosed with advanced gastric cancer in his 40s. Within months, the patient died of digestive tract obstruction. III:6 was diagnosed with gastric stromal cancer at 56 years. She underwent exploratory laparotomy, which revealed a large ruptured mass in the greater curvature of the stomach, and finally underwent partial gastrostomy. The patient died 1 year after surgery due to extensive metastasis. At the time of recruitment, Patients III:5 and III:6 had passed away over 30 and 10 years prior, respectively. Therefore, blood samples were not available.

Two patients were diagnosed with intestinal adenocarcinomas. III:3 was diagnosed with duodenal adenocarcinoma using gastroscopy at 53 years. The patient underwent a segmental resection after diagnosis. At the time of recruitment, she was in good health and showed no signs of disease progression during annual examinations. III:3 underwent WES after recruitment, which revealed a heterozygous *XAF1* Mut (c.454+1372G>A). Sanger sequencing was used to validate these results (Figure 1B). III:1 was diagnosed with poorly differentiated colon adenocarcinoma by colonoscopy biopsy of a mass at the hepatic flexure at 58 years. In addition, magnetic resonance imaging suggested bilateral metastases to the iliac bones. The patient was admitted with FOLFOX4 (5‐fluorouracil, leucovorin, and oxaliplatin) treatment. After diagnosis by colonoscopy, the patient survived for more than 6 months. Genotype III:1 was uncertain because no blood samples were available for testing.

Two patients in Generation III were diagnosed with hepatocellular cancer. At the age of 46 years, III:9 was diagnosed with hepatocellular carcinoma with small‐cell histology, a rare morphological variant of hepatocellular cancer, after undergoing liver transplantation. The patient was under surveillance for 14 years, with annual examinations until enrollment. Genotype III:9 was identified as a heterozygous variant of *XAF1* (c.454+1372G>A) by WES and bidirectional Sanger sequencing (Figure 1C). III:4 was diagnosed with hepatocellular carcinoma in her 60s. Due to poor economic conditions, she was unable to access appropriate treatment. She had died over 10 years prior to the time of enrollment. Therefore, the genotype was not determined in this study.

Four individuals in Generation III were cancer‐free at the time of enrollment. The patients were aged 65 (III:2), 61 (III:7), 55 (III:8), and 50 years (III:11) at the time of enrollment. The results of WES and Sanger sequencing showed that Genotypes III:2 and III:7 were both homozygous for wild‐type XAF1, and Genotype III:11 was a heterozygous variant of *XAF1* (c.454+1372G>A).

### 2.3. Germline Mut (c.454+1372G>A) of *XAF1* Was Found at Increased Frequency in GI Cancer Patients

Considering the observation of loss of function (LOF) variant in tumor suppressor gene *XAF1* in Exon 4b (c.454+1372G>A) among three GI cancer patients in the family with high GI cancer incidence and the very low frequency of this single nucleotide polymorphism (SNP) in the databases, we decided to explore the frequency of this variant in a set of 148 Chinese Han samples with unselected GI cancers, including 95 gastric cancer patients, 53 esophageal cancer patients, and 283 cancer‐free controls.

Table 2 presents the demographic characteristics of the participants, including smoking and drinking habits. The mean age of patients with esophageal cancer (65.70 ± 7.627 years, *t*‐test; *p* value 0.009) and gastric cancer (62.70 ± 10.190 years, *t*‐test; *p* value < 0.001) was older than that of controls (59.32 ± 11.307 years). The risk of esophageal and gastric cancers increased with increased cigarette consumption (chi‐square linear‐by‐linear association; both *p* value < 0.001). In addition, the risk of esophageal and gastric cancers increased with increased alcohol consumption (chi‐square linear‐by‐linear association; both *p* value < 0.001).

**Table 2:** Demographic characteristics of individuals in the gastric cancer, esophageal cancer, and nontumor groups.

	Esophageal cancer patients	Gastric cancer patients	Cancer‐free controls
No.	53	95	283
Age, years, mean (SD)	65.70 (7.627)^a^	62.74 (10.190)^b^	59.32 (11.307)
BMI, kg/m^2^, mean (SD)	22.54 (3.122)	22.68 (2.923)	23.429 (3.059)
Sex, male (%)	51 (96.2%)^c^	67 (70.5%)^d^	124 (43.8%)
Smoking			
Abstainer	16 (30.2%)^e^	52 (54.7%)^e^	220 (77.7%)
Sporadically	0 (0.0%)^e^	3 (3.2%)^e^	10 (3.5%)
Regular consumption			
0 < pack − years ≤ 20	11 (20.8%)^e^	10 (10.5%)^f^	23 (8.1%)
20 < pack − years ≤ 40	19 (35.8%)^e^	23 (24.2%)^f^	25 (8.8%)
40 < pack − years	7 (13.2%)^e^	7 (7.4%)^f^	5 (1.8%)
Alcohol consumption			
Abstainer	17 (32.1%)^g^	58 (61.1%)^h^	221 (78.1%)
Sporadically	14 (26.4%)^g^	9 (9.5%)^h^	37 (13.1%)
Regular intake			
0 < years ≤ 20	5 (9.4%)^g^	4 (4.2%)^h^	9 (3.2%)
20 < years ≤ 40	14 (26.4%)^g^	16 (16.8%)^h^	11 (3.9%)
40 < years	3 (5.7%)^g^	8 (8.4%)^h^	5 (1.8%)
Family history of cancer	4 (7.5%)	10 (10.5%)	7 (2.5%)
Multiple primary cancers	3 (5.7%)	5 (5.3%)	/

^a^ *p* value < 0.001 (esophageal cancer vs. controls) (age).

^b^ *p* value = 0.009 (gastric cancer vs. controls) (age).

^c^ *p* value < 0.001 (esophageal cancer vs. controls) (sex).

^d^ *p* value < 0.001 (gastric cancer vs. controls) (sex).

^e^ *p* value < 0.001 (esophageal cancer vs. controls) (smoking).

^f^ *p* value < 0.001 (gastric cancer vs. controls) (smoking).

^g^ *p* value < 0.001 (esophageal cancer vs. controls) (drinking).

^h^ *p* value < 0.001 (gastric cancer vs. controls) (drinking).

The genotype and frequencies of *XAF1* variation in Exon 4b (c.454+1372G>A) in patients with esophageal and gastric cancer and controls are shown in Table 3. No homozygous Mut of c.454+1372G>A was observed among GI cancer patients and controls, or among members of the participating family. Among the 283 cancer‐free individuals, only 1 (0.35%) had a heterozygous Mut in *XAF1* (c.454+1372G>A). The allele frequency reported in databases is relatively low, 0.00006 in the ExAC database, 0.0009 in the 1000 Genomes Project database, and 0.000185 in the gnomAD database, as was described above. According to data from the 1000 Genomes Project, the allele frequencies within Chinese subpopulations were as follows: 0.004902 in the Han Chinese from Beijing, 0.004902 in the Southern Han Chinese, and 0.000 in the Dai ethnic group from Xishuangbanna. A heterozygous Mut in *XAF1* (c.454+1372G>A) was detected in six (4.05%) patients with GI cancer, consisting of three (5.66%) esophageal cancer patients and three (3.16%) gastric cancer patients. After adjusting for confounding factors, age, smoking, and drinking habits, logistic regression analysis supported the finding that the *XAF1* Mut was associated with GI cancer (odds ratio [OR] = 9.293; 95% confidence interval [95*%*CI] = 1.016–85.009; *p* value = 0.049). A significantly increased risk of esophageal cancer was found with a heterozygous Mut in *XAF1* (OR = 18.301; 95% CI = 1.204–278.166; *p* value = 0.036). For gastric cancer, logistic regression analysis revealed an increased risk of heterozygous Muts in *XAF1* (OR = 7.456; 95% CI = 0.732–75.950; *p* value = 0.090). However, the result was not statistically significant.

**Table 3:** Validation of germline *XAF1* mutation (c.454+1372G>A) by Sanger sequencing in gastric cancer, esophageal cancer, and nontumor groups.

*XAF1* genotypes^a^	Controls	GI cancers				Esophageal cancers				Gastric cancers			
	*n*	*n*	OR^b^	95%CI^b^	*p* ^b^	*n*	OR^c^	95%CI^c^	*p* ^c^	*n*	OR^d^	95%CI^d^	*p* ^d^
−/−	282/283 (99.65%)	142/148 (95.95%)				50/53 (94.34%)				92/95 (96.84%)			
+/−	1/283 (0.35%)	6/148 (4.05%)	**9.293**	**1.016–85.009**	**0.049**	3/53 (5.66%)	**18.301**	**1.204–278.166**	**0.036**	3/95 (3.16%)	7.456	0.732–75.950	0.090

*Note:* Data for gastric cancer, esophageal cancer, and control groups are presented as *n* or *n* (%). *p*<0.05 are indicated in boldface.

^a^Compared with noncancer controls. *XAF1* genotypes are classified based on the SNP mutation of c.454+1372G>A, −/− refers to wildtype, and +/− refers to heterozygous mutation. None +/+ homozygous mutation of c.454+1372G>A was identified in this case‐control study.

^b^Analysis of genotype with adjusted values for confounding factors (age, smoking, and drinking habits) in logistic regression.

### 2.4. Reduced XAF1 Protein Expression and Increased XIAP Protein Expression in Patients With XAF1 Heterozygous Mut (c.454+1372G>A)

**Figure 2:**
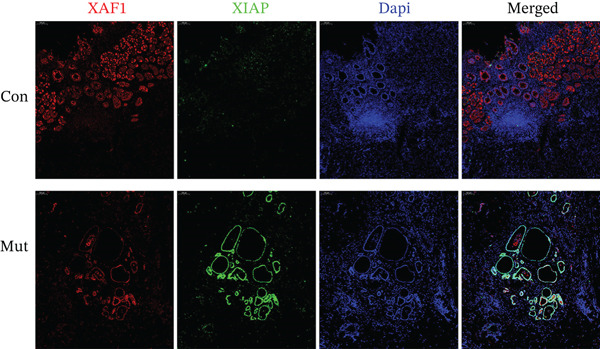
Immunofluorescence analysis of XAF1 and XIAP in human gastric cancer tumor tissue. XAF1 expression is decreased in the mutation group compared to the control group, while XIAP expression is increased. Mut: patients with gastric cancer exhibiting the XAF1 heterozygous mutation; Con: patients with gastric cancer without the XAF1 heterozygous mutation.

To determine whether the *XAF1* heterozygous Mut leads to abnormal expression of the XAF1 protein, we performed immunofluorescence staining for XAF1 protein in tumor tissue sections obtained from patients with GI tumors. As shown in Figures S2 and S3, XAF1 was mainly expressed in epithelial tissues, including the gastric mucosal epithelium and esophageal squamous epithelium. In gastric cancer tumor tissues, XAF1 was abundantly expressed in glandular cells (Figure 2). Furthermore, XAF1 expression was located mainly in the cytoplasm, especially around the nucleus, whereas XIAP, which is an antagonist of XAF1, was expressed mainly in the nucleus.

Immunofluorescence revealed reduced expression of XAF1 in gastric adenocarcinoma in GC patients harboring a heterozygous Mut compared to that in GC patients without the Mut (Con), as shown in Figure 2. XIAP expression was higher in patients with Mut‐GC than in those with Con‐GC.

**Figure 3:**
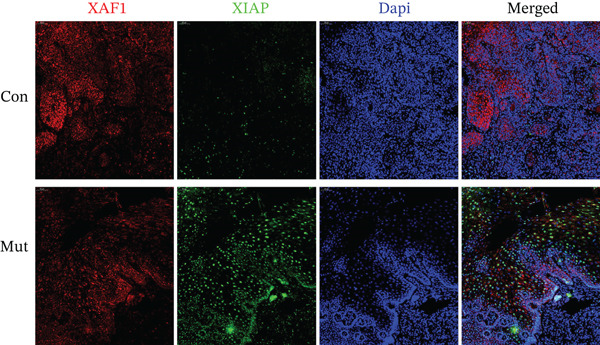
Immunofluorescence analysis of XAF1 and XIAP in human esophageal cancer tumor tissue. XAF1 expression is decreased in the mutation group compared to the control group, while XIAP expression is increased. Mut: patients with esophageal cancer exhibiting the *XAF1* heterozygous mutation; Con: patients with esophageal cancer without the *XAF1* heterozygous mutation.

In esophageal squamous cell carcinoma, reduced XAF1 expression was observed in Mut‐EC patients compared to Con‐EC patients (Figure 3). Consistent with findings in patients with GC, the expression of XIAP was higher in Mut‐EC than in Con‐EC. Furthermore, XAF1 expression was observed in the cytoplasm surrounding the nucleus, whereas XIAP expression was limited to the nucleus in only a few cells. The areas with the more XAF1 expression were accompanied by the lower XIAP expression (Figure 3, Con). XIAP expression in Mut‐EC patients was not limited to the nucleus and was also observed in the cytoplasm (Figure 3, Mut).

XAF1 expression results from altered DNA methylation in the promoter region. We analyzed the DNA methylation patterns in these two groups, utilizing peripheral blood cells from five patients with GI cancer with the *XAF1* Mut and nine patients without the Mut. For XAF1, methylation levels across the 16 DMPs in these two groups were comparable, as shown in Figure S4a. Similarly, in XIAP, there was no significant difference in methylation levels across the 19 DMPs, as shown in Figure S4b.

### 2.5. Somatic Second‐Hit Changes

In order to validate the second hit in somatic cells, we compared the methylation status of the *XAF1* promoter region between patients with GI cancer and cancer‐free individuals. As shown in Figure S4c, methylation levels at two CpG sites, cg22116016 and cg03419151, were found to be significantly higher in patients with GI cancer (FDR *q* values = 0.019 and 0.031, respectively). Formalin‐fixed, paraffin‐embedded (FFPE) tumor tissue sections were obtained from four patients with GI cancer harboring *XAF1* c.454+1372G>A Mut. After extracting the genomic DNA from these specimens, we performed WES and LOH analysis to compare tumor tissues with matched adjacent normal tissues. As shown in Figure S5, among these three patients, two exhibited LOH within the region containing *XAF1* on Chromosome 17 (specifically in Regions 6005–10,419,661 and 1,303,262–8,248,797, respectively). The remaining patient maintained a copy number‐neutral status in the *XAF1*‐containing region (4,897,600–7,724,689) on Chromosome 17.

### 2.6. Reduced Apoptosis Was Observed in Tumor Tissues of Patients With Heterozygous *XAF1* Muts

**Figure 4:**
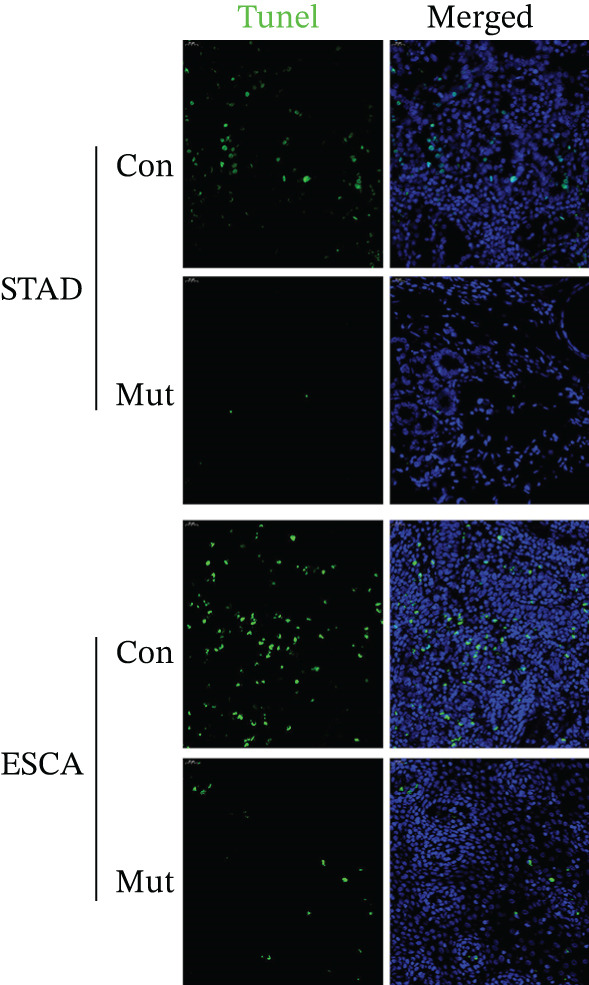
TUNEL assay of gastric and esophageal cancer tissue. The level of apoptosis was lower in the groups with heterozygous *XAF1* mutations compared to the wild‐type genotype groups for both gastric and esophageal cancer.

To determine whether increased XIAP expression caused by heterozygous Muts in *XAF1* further inhibits apoptosis, we conducted a terminal deoxynucleotidyl transferase deoxyuridine triphosphate nick‐end labeling (TUNEL) assay. As anticipated, a higher number of stained cells were detected in patients with gastric cancer exhibiting the wild‐type *XAF1* genotype than in those with heterozygous Muts. This suggests that apoptosis is inhibited in those with heterozygous *XAF1* Muts. Similarly, in patients with esophageal cancer, the level of apoptosis was lower in patients with heterozygous *XAF1* Muts than in those with the wild‐type genotype (Figure 4).

## 3. Discussion

WES analysis of the family with high GI cancer incidence identified a LOF variant in the tumor suppressor gene *XAF1* (c.454+1372G>A, minor allele frequency [MAF] < 0.01), producing a stop codon in Exon 4b and a truncated protein of XAF1 Isoform 5. Moreover, a higher frequency of this SNP in *XAF1* (c.454+1372G>A) was detected in patients with esophageal and gastric cancers, revealing that the SNP is associated with an increased risk of these two GI cancers.

XAF1 has been proposed as a putative tumor suppressor that induces apoptosis, a crucial and complex process implicated in the elimination of malignant cells. Liston et al. first identified XAF1 as a ZF protein that inhibits the antiapoptotic activity of XIAP [29]. Increased promoter methylation and *XAF1* silencing have been observed in various GI cancers, including gastric adenocarcinoma [18, 21], colon cancer [24, 30, 31], esophageal cancer [32], and liver cancer [33, 34]. Restoration of XAF1 expression enhances TRAIL‐induced apoptosis and inhibits tumor progression [35]. Moreover, the missense SNP rs34195599 is associated with the development of papillary thyroid cancer [25]. Pinto et al. discovered an extended haplotype harboring both TP53‐R337H and XAF1‐E134∗ Muts that was significantly enriched in patients with cancer compared with newborns [22]. Subsequent promoter–reporter studies have demonstrated that XAF1 functions as a physiological modulator of p53. Taken together, these findings suggest that genetic variants of *XAF1* are risk factors for cancer development.

Given that the expression of XAF1 is involved in tumor progression and that the above two SNPs of *XAF1* are associated with cancer predisposition, it is reasonable to propose that the SNP of *XAF1* (c.454+1372G>A) in the family with high GI cancer incidence could be related to GI cancer predisposition. Genetic screening of 283 cancer‐free controls with a mean age of 59.32 years revealed only one participant (male, 57 years) who harbors the SNP of *XAF1* (c.454+1372G>A). However, six positive carriers (three with gastric cancer and three with esophageal cancer) were detected among 148 total patients with GI cancer. We found that the heterozygous SNP of *XAF1* (c.454+1372G>A) was associated with a significantly increased GI cancer risk (OR = 9.293, 95% CI = 1.016–85.009, *p* value = 0.049). Eastern Asia has the highest lifetime risk of stomach and esophageal cancers globally. Notably, the allele frequency of this SNP of *XAF1* (c.454+1372G>A) was reported to be the highest in the eastern Asian population (0.0050 in the 1000 Genomes Project database) and was barely detected in the African European and American populations (0.000 in the 1000 Genomes Project database). This genetic variant in *XAF1* may partially explain the high risk of GI cancer in East Asia. The etiology of GI tumors is believed to result from the interaction between genetic and environmental factors. In the present study, a higher incidence of regular alcohol consumption was observed in patients with esophageal cancer (41.5%) and gastric cancer (29.4%) than in cancer‐free controls (8.9%). Moreover, the rate of regular tobacco use was significantly higher in patients with GI cancer (69.8% in patients with esophageal cancer and 42.1% in patients with gastric cancer) than in cancer‐free controls (18.7%). After adjusting for alcohol and tobacco consumption and age, logistic regression analysis revealed that polymorphisms in Exon 4b of *XAF1* were significantly associated with susceptibility to GI cancer. However, when we compared the risk of gastric and esophageal cancers separately with cancer‐free controls, the results were inconsistent. Our data demonstrated that the SNP of *XAF1* (c.454+1372G>A) had a significant promotive effect on esophageal cancer, whereas the promotive effect on gastric cancer was not significant (OR 7.456, 95% CI 0.732–75.950, *p* = 0.090). This may be due to the relatively low frequency of the *XAF1* SNP (c.454+1372G>A) and the limited sample size. Accordingly, a cohort study is needed to compare the clinical outcomes between groups with heterozygous Muts in *XAF1* (c.454+1372G>A) and wild‐type *XAF1*.

XAF1 regulates apoptosis and tumorigenesis by repressing XIAP expression. Transcriptional repression of XAF1 disrupts the intracellular balance of XIAP and XAF1, resulting in the upregulation of XIAP [36]. Moreover, XAF1 overexpression has been shown to suppress XIAP expression in epithelial ovarian cancer cells [37]. Our study demonstrated that the expression of XAF1 in cancer patients with a heterozygous Mut in *XAF1* (c.454+1372G>A) was lower than that in cancer patients with wild‐type *XAF1*. Meanwhile, the expression of XIAP was upregulated in patients with the heterozygous *XAF1* Mut, which is consistent with previous studies.

A previous study suggested that allelic loss in tumor cells leads to decreased XAF1 expression, suggesting a gene dose effect of *XAF1* alleles [19]. It is well‐established that aberrant DNA methylation in the promoter region leads to the transcriptional inactivation of *XAF1* in tumors [21, 32, 38–40]. Previous studies have suggested that peripheral blood DNA methylation may serve as a suitable biomarker for cancer patients, with *XAF1* methylation in particular being associated with gastric cancer prognosis (R). Peripheral blood analysis showed hypermethylation at two *XAF1* promoter region CpG sites in GI cancer compared to controls. Additionally, LOH was observed in tumor tissues at a high rate (two out of three cases). However, owing to the limitations of cancer tissues from patients harboring the heterozygous Mut of *XAF1* (c.454+1372G>A), we did not detect methylation levels in the tissues obtained for this study. In the future, a prospective cohort study using cancer tissues from patients harboring the heterozygous Mut of *XAF1* (c.454+1372G>A) should be conducted alongside methylation analysis and western blotting to further validate the second‐hit epigenetic effect during tumorigenesis.

*XAF1* c.454+1372G>A Mut identified in this study constitutes a LOF Mut in Isoform 5. Previous studies have reported increased expression of truncated XAF1 isoforms in tumor tissues. Such findings suggest that the site of interaction between XAF1 and XIAP was located in the CTD within the amino acid range of 214–301 in full‐length XAF1 [41], specifically in the ZF7 domain. Coincidentally, XAF1 Isoform 5 mainly comprises ZF6 and ZF7, indicating a potential interaction between XAF1 Isoform 5 and XIAP. Our study showed that the expression of XAF1 Isoform 5 was significant in normal gastric and esophageal tissues. Consequently, the nonsense Mut in XAF1 Isoform 5 (c.454+1372G>A) can result in a premature stop codon in Exon 4b and reduce the expression of XAF1 Isoform 5, resulting in decreased suppression of XIAP.

This study reveals a novel genetic spectrum of GI cancer, and to our knowledge, this is the first study to report an association between GI cancer and *XAF1* c.454+1372G>A. Our findings suggest that *XAF1* c.454+1372G>A may contribute to reduced XAF1 expression and increased XIAP expression, with reduced apoptosis in tumor tissues. Further studies on the *XAF1* c.454+1372G>A cohort will enhance our understanding of the effects of germline *XAF1* Muts and have significant implications for genetic counseling and the clinical management of carriers.

## 4. Materials and Methods

### 4.1. Ethics Statement

This research project was approved by the Ethics Committee of the Second Affiliated Hospital of Zhejiang University School of Medicine. All participants provided informed consent for the reporting and publication of their individual patient data.

### 4.2. Study Subjects

A Chinese pedigree with GI cancer was recruited for this study. Of the 11 members of the third generation, seven were diagnosed with GI cancers, six of whom were diagnosed before 60 years of age. At the time of the study, seven members of the third generation were alive. Of them, six volunteered for peripheral blood and clinical data collection.

### 4.3. Case‐Control Study

All patients with a clinical and histopathological diagnosis of gastric and esophageal cancer who were treated at the Department of Surgery, Second Affiliated Hospital of Zhejiang University School of Medicine, from May 1, 2022, to December 31, 2023, were eligible for participation. Cancer‐free individuals aged 40–80 years undergoing annual physical examinations were invited to participate as a control group. Detailed baseline characteristics included demographic data, age at diagnosis, body mass index, family history of cancer, any other type of primary cancer, smoking, and alcohol consumption.

Participants were asked about the average number of cigarettes consumed per day before the diagnosis of GI cancer and their years of smoking. Based on self‐reported information, smoking patterns were classified into three groups: subjects who achieved more than 1 year of abstinence before diagnosis (abstainer), occasional smokers (sporadic), and daily smokers (daily). For daily smokers, pack‐years were calculated by multiplying the average number of cigarettes consumed per day by the years of smoking and dividing by 20. Alcohol intake patterns were classified into three categories: abstainer, sporadic, and daily. The duration of regular alcohol intake before the diagnosis of GI cancer was recorded for daily consumers. Each cancer group was compared to the control group using ORs. Comparisons of continuous variables between groups were performed using *t*‐tests. Comparisons of ordinal categorical variables were performed using chi‐square linear‐by‐linear association.

### 4.4. WES and Data Analysis

Qualified genomic DNA samples were randomly fragmented using Covaris technology, resulting in library fragments predominantly between 150 and 250 bp in size. The DNA fragments underwent end repair, and an “A” base was added to the 3 ^′^‐end of each strand. Adapters were ligated to both ends of the end‐repaired/dA‐tailed DNA fragments to prepare them for amplification and sequencing. The size‐selected DNA fragments were amplified using ligation‐mediated PCR (LM‐PCR), purified, and hybridized to an exome array for enrichment. Nonhybridized fragments were washed away, and the captured products were circularized. Rolling circle amplification was performed to produce DNA nanoballs (DNBs). The exome‐captured libraries were loaded onto the DNBSEQ sequencing platform (BGI, China). All sequencing data were aligned to the human genome reference (build hg38) using the Burrows–Wheeler Aligner (BWA), duplicate reads were marked using Picard, and base quality score recalibration was performed using the Genome Analysis Toolkit. Germline single nucleotide variants (SNVs) and insertions and deletions (indels) were identified using the GATK HaplotypeCaller. We annotated variations using ANNOVAR. We filtered the variants against the 1000 Genomes Project, the Exome Variant Server (ESP6500), and the ExAC to exclude variants with global MAF ≥ 0.01. In addition, variants with MAF ≥ 0.01 in the East Asian subpopulation were also filtered. We exclusively focused on protein‐altering variants, including nonsense SNVs, missense SNVs, splicing‐site SNVs, and frameshift indels. The potentially damaging effects of missense SNVs were predicted using SIFT and PolyPhen‐2. The most severe effect of the variants was observed during curation.

### 4.5. Sanger Sequencing

The following primers for the identified variations in *XAF1* were designed using Primer‐BLAST (http://www.ncbi.nlm.nih.gov/tools/primer-blast) and synthesized by Sangon Biotech (Shanghai, China): forward primer (5 ^′^‐GAGCACAGTCAGAATGGAGGG‐3 ^′^) and reverse primer (5 ^′^‐ATGGGACCCTTTAATATCCATGCT‐3 ^′^). After bidirectional PCR amplification, the reaction products from 6 pedigree members, 283 cancer‐free controls, and 148 patients with GI cancer (95 gastric cancer and 53 esophageal cancer) were sequenced by Sanger sequencing on an ABI 3100 DNA analyzer (Applied Biosystems, Foster City, California, United States). Binary logistic regression analysis was used to predict associations between the identified variations in XAF1 expression and susceptibility to GI cancers. Participants with “sporadic” and “abstainer” smoking statuses were integrated into one group because the number of sporadic smokers in esophageal cancers was 0, which could not be analyzed. Association was expressed as ORs for risk estimation at 95% CIs. All statistical analyses were performed using SPSS software (Version 27.0; SPSS, Chicago, Illinois, United States).

### 4.6. Immunofluorescence

Sections of surgically resected tumors were obtained from three patients with esophageal cancer and three patients with gastric cancer identified as *XAF1* heterozygous mutants. Sections from three patients with esophageal cancer and three patients with gastric cancer without genetic variations in *XAF1* were collected as nonmutant controls. All sections were obtained from the pathology department after obtaining patient consent. Normal esophageal and stomach tissues were collected after total gastrectomy and esophagectomy from two volunteer patients without the *XAF1* Mut. Immunofluorescence was performed to evaluate the protein expression of XAF1 and XIAP. The tissue specimens were fixed with 4% formaldehyde, permeabilized with 100% methanol, and blocked with 0.1% Triton X‐100. The sections were then incubated overnight at 4°C with anti‐XAF1 antibody (ab81353, Abcam) and mouse anti‐XIAP antibody (130‐10448‐20, RayBiotech). After washing with PBS, sections were incubated with secondary antibodies for 2 h at room temperature. Nuclei were stained with DAPI (1 *μ*g/mL, Sigma). Negative controls included parallel sections treated without the primary antibody. The slides were analyzed using an ECLIPSE C1 fluorescence microscope (NIKON, Tokyo, Japan).

### 4.7. TUNEL Staining

Apoptosis in surgically resected cancer tissues was determined by TUNEL assay using the One‐Step TUNEL Apoptosis Assay Kit (cat. no. C1086, Beyotime, China), according to the manufacturer’s instructions. In the TUNEL assay, the terminal deoxynucleotidyl transferase was used to add fluorescein isothiocyanate (FITC)–labeled dUTP to the 3 ^′^ ends of fragmented DNA in apoptotic cells. Sections were visualized under a fluorescence microscope (NIKON, Tokyo, Japan).

### 4.8. Immunoblot of XAF1

Total soluble proteins were extracted from normal gastric and esophageal tissues using RIPA buffer. A polyclonal antibody (ab81353, Abcam) was used for XAF1 detection at a concentration of 1 *μ*g/mL. Horseradish peroxidase‐conjugated antirabbit IgG was used as a secondary antibody.

### 4.9. MethylationEPIC v2.0 (935 K) BeadChip and Bioinformatic Analysis

To evaluate DNA concentration and maintain its integrity, we used Qubit 4.0 (Invitrogen) and agarose gel electrophoresis, respectively. Subsequently, DNA was subjected to bisulfite treatment. The converted DNA was then subjected to analysis on an Illumina Infinium MethylationEPIC v2.0 (935 K) BeadChip platform (Illumina). Subsequently, the chip was scanned using an Illumina iSCAN system to obtain the Idat files. These files were imported into R and preprocessed using the SESAME package (Version 1.20.0) to extract raw data. Statistical comparisons between the two groups for continuous variables were performed using the *F*‐test. To identify significantly differentially methylated loci, we applied a threshold of |log2*F* *C*| > 1 and a *p* value of < 0.05. For a given contrast, neighboring CpGs that showed consistent methylation variations were merged into differentially methylated regions, and we applied a threshold of *p* < 0.05.

### 4.10. DNA Extraction From FFPE Sections and Subsequent LOH Analysis

All of the tumor tissue had been surgically resected, fixed in 10% neutral‐buffered formalin, embedded in paraffin, in the course of routine surgical procedures, and was surplus to diagnostic requirements. For each patient, 10 sections of tumor tissue and 10 sections of adjacent nontumor tissue (devoid of tumor infiltration) were collected to form a matched‐pair control set. Genomic DNA was extracted from each sample. Libraries for exome sequencing were constructed from isolated DNA. WES was performed as previously described. We employed Control‐FREEC for copy number variation (CNV) detection, which statistically infers CNV types based on the depth distribution of reads aligned to the reference genome.

**Nomenclature**
GI: gastrointestinal
IF: immunofluorescence
XAF1: X‐linked inhibitor of apoptosis protein associated factor 1
XIAP: X‐linked inhibitor of apoptosis protein
WES: whole exome sequencing
ZF: zinc finger
NTD: N‐terminal domain
MD: middle domain
CTD: C‐terminal domain
GRP78: glucose‐regulated protein 78
XELOX: oxaliplatin and capecitabine
CT: computed tomography
FOLFOX4: 5‐fluorouracil, leucovorin, and oxaliplatin
SNP: single nucleotide polymorphism
GC: gastric adenocarcinoma
EC: esophageal squamous cell carcinoma
Mut: mutation
Con: control
TUNEL: terminal deoxynucleotidyl transferase dUTP nick end labeling
TRAIL: tumor necrosis factor (TNF)–related apoptosis‐inducing ligand
OR: odds ratio
CI: confidence interval
DNA: deoxyribonucleic acid
BMI: body mass index
LM‐PCR: ligation‐mediated polymerase chain reaction
DNB: deoxyribonucleic acid nanoball
SNV: single nucleotide variant
MAF: minor allele frequency
PBS: phosphate‐buffered saline
DAPI: 4 ^′^,6‐diamidino‐2‐phenylindole
FITC: fluorescein isothiocyanate

## Author Contributions

Min Jin and Dan‐Qing Yu conceived and designed the study. Guan‐Xin Xu and Hang Zhang were responsible for patient enrollment and follow‐up. Chang‐Xing Wang performed pathological analysis of the surgically resected specimens. Ying‐Zhi Zhang carried out the collection and analysis of blood samples. Yao Ning and Miao Shen conducted DNA extraction from blood samples and subsequent validation by Sanger sequencing. Ping‐Ping Lv and Chun Feng were in charge of data analysis. Guan‐Xin Xu performed the genotyping and methylation analysis. Guan‐Xin Xu and Dan‐Qing Yu wrote the first draft of the manuscript. All authors contributed to the interpretation of the results and critically revised the manuscript for important intellectual content. Guan‐Xin Xu and Hang Zhang should be regarded as joint first authors.

## Funding

The study was funded by the National Natural Science Foundation of China (10.13039/501100001809, 81901498).

## Disclosure

All authors approved the final version for submission.

## Conflicts of Interest

The authors declare no conflicts of interest.

## Supporting Information

Additional supporting information can be found online in the Supporting Information section.

## Supporting information


**Supporting Information 1** Figure S1. The Western blot of various XAF1 protein isoforms in normal esophageal and gastric tissues. The Western blot results showed multiple band patterns for the XAF1 antibody, suggesting the expression of various XAF1 protein isoforms.

**Supporting Information 2** Figure S2. XAF1 expression in normal gastric tissue. Immunofluorescence staining shows high expression of XAF1 in the normal gastric mucosal epithelium. (a) HE stain; (b) XAF1 expression shown as red fluorescence; (c) DAPI staining shown as blue fluorescence.

**Supporting Information 3** Figure S3. XAF1 expression in normal esophageal tissue. Immunofluorescence staining demonstrated high expression of XAF1 in normal esophageal tissue. (a) HE stain; (b) XAF1 expression shown as red fluorescence; (c) DAPI staining shown as blue fluorescence.

**Supporting Information 4** Figure S4. DNA methylation levels of *XAF1* and *XIAP* genes. (a) DNA methylation patterns in the promoter region of the *XAF1* gene. Blue: GI cancer patients carrying the *XAF1* mutation (c.454+1372G>A). Orange: GI cancer patients without this mutation. (b) DNA methylation patterns in the promoter region of the XIAP gene. Blue: GI cancer patients carrying the *XAF1* mutation (c.454+1372G>A). Orange: GI cancer patients without this mutation. (c) DNA methylation patterns in the promoter region of the *XAF1* gene. Blue: cancer‐free individuals carrying the *XAF1* mutation (c.454+1372G>A). Orange: GI cancer patients with this mutation.

**Supporting Information 5** Figure S5. Copy number variations (CNVs) of Chromosome 17. The distribution of copy number variations across Chromosome 17 in three patients. Blue: decreased copy number; green: copy number neutral; red: increased copy number.

**Supporting Information 6** Table S1. Alternative splicing of the *XAF1* transcript and isoforms of the XAF1 protein. NMD, nonsense‐mediated mRNA decay.

## Data Availability

The data that support the findings of this study are available on request from the corresponding authors. The data are not publicly available due to privacy or ethical restrictions.
