# Supplementary figures and images for: Identification of a Germline XAF1 Mutation in Patients With Gastrointestinal Cancers
Source: Hum Mutat. 2026 May 4;2026:4279712. doi: 10.1155/humu/4279712 (PMC13139716; doi:10.1155/humu/4279712)

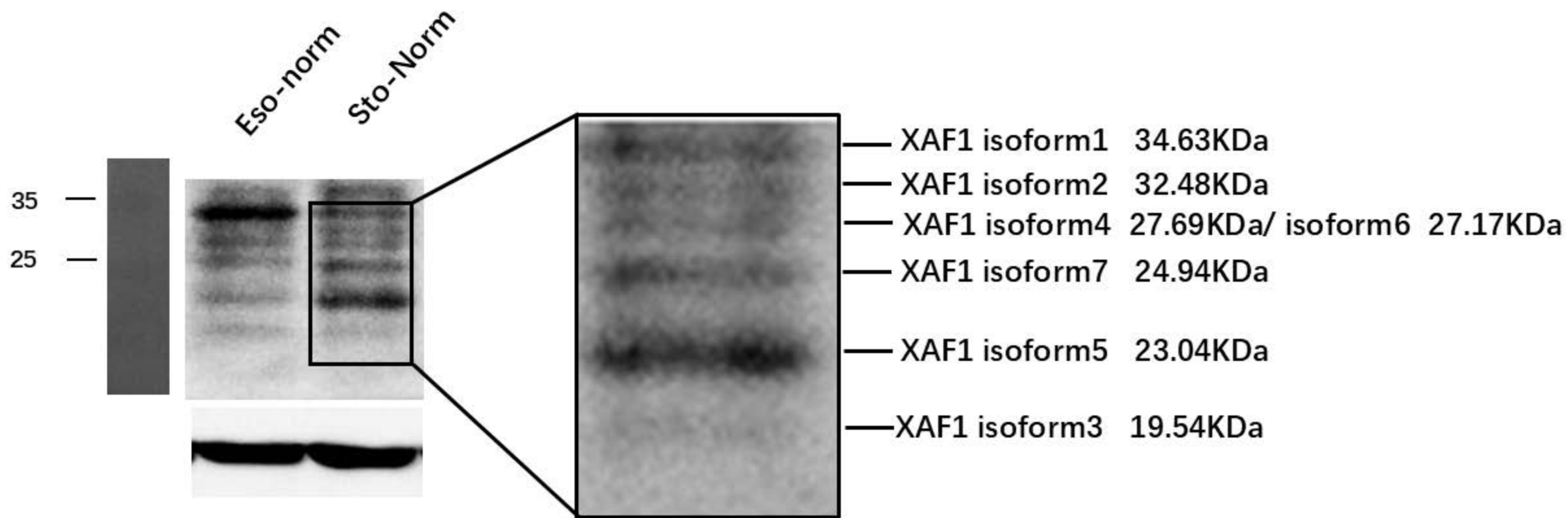

Supplement: Supplementary file 1 — Supporting Information 1 Figure S1. The Western blot of various XAF1 protein isoforms in normal esophageal and gastric tissues. The Western blot results showed multiple band patterns for the XAF1 antibody, suggesting the expression of various XAF1 protein isoforms. [file HUMU-2026-4279712-s006.pdf]

## XAF1 expression in normal gastric tissue

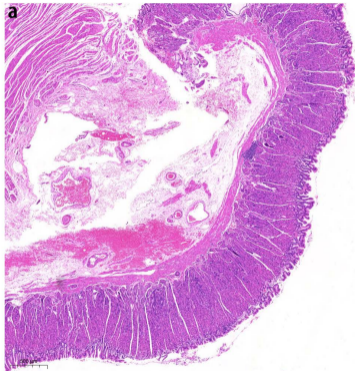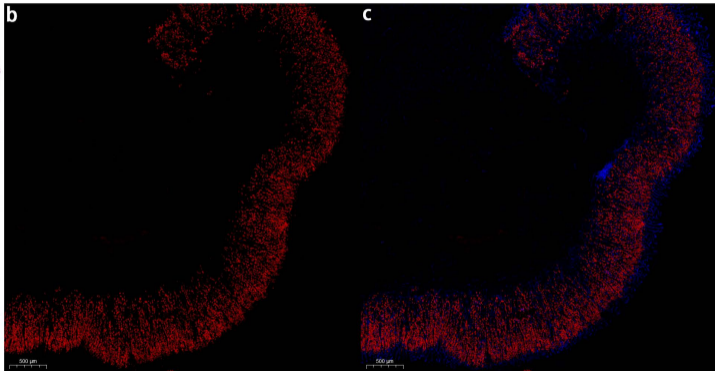

Supplement: Supplementary file 2 — Supporting Information 2 Figure S2. XAF1 expression in normal gastric tissue. Immunofluorescence staining shows high expression of XAF1 in the normal gastric mucosal epithelium. (a) HE stain; (b) XAF1 expression shown as red fluorescence; (c) DAPI staining shown as blue fluorescence. [file HUMU-2026-4279712-s004.pdf]

## XAF1 expression in normal esophageal tissue

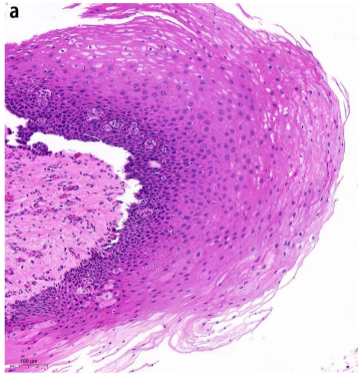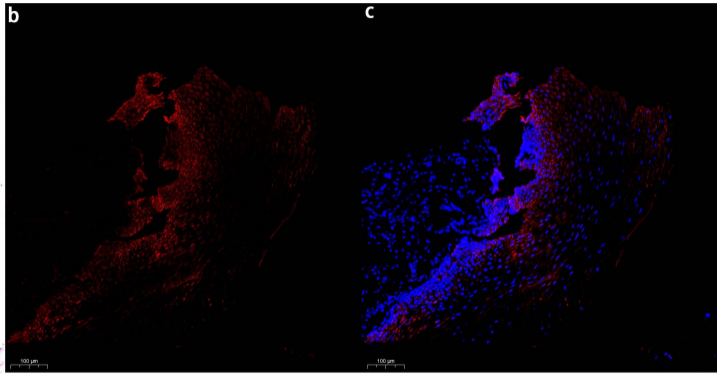

Supplement: Supplementary file 3 — Supporting Information 3 Figure S3. XAF1 expression in normal esophageal tissue. Immunofluorescence staining demonstrated high expression of XAF1 in normal esophageal tissue. (a) HE stain; (b) XAF1 expression shown as red fluorescence; (c) DAPI staining shown as blue fluorescence. [file HUMU-2026-4279712-s003.pdf]

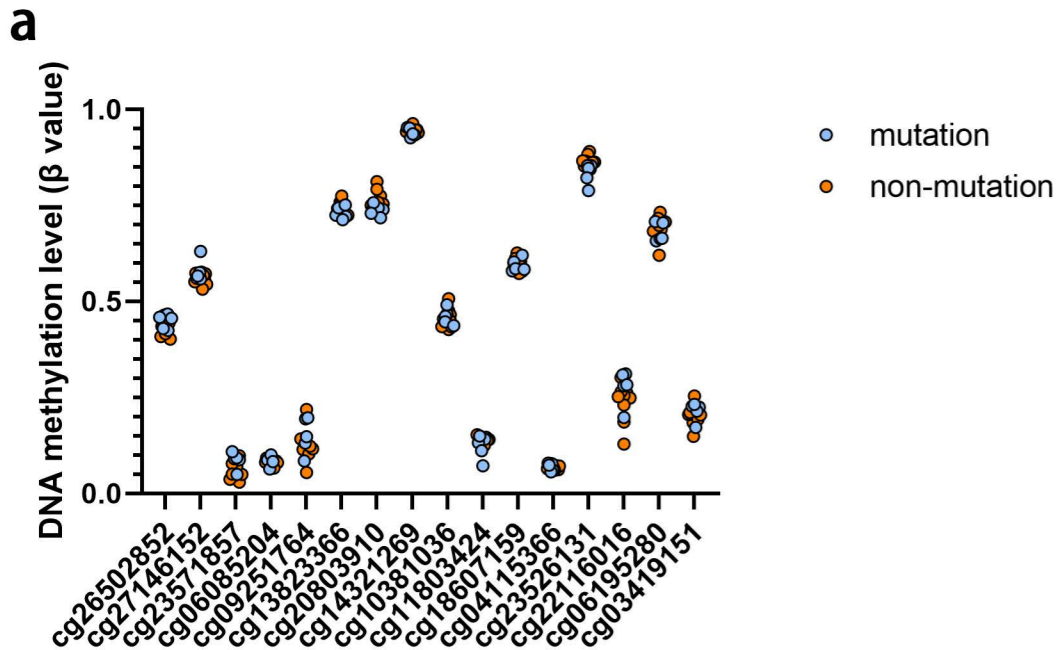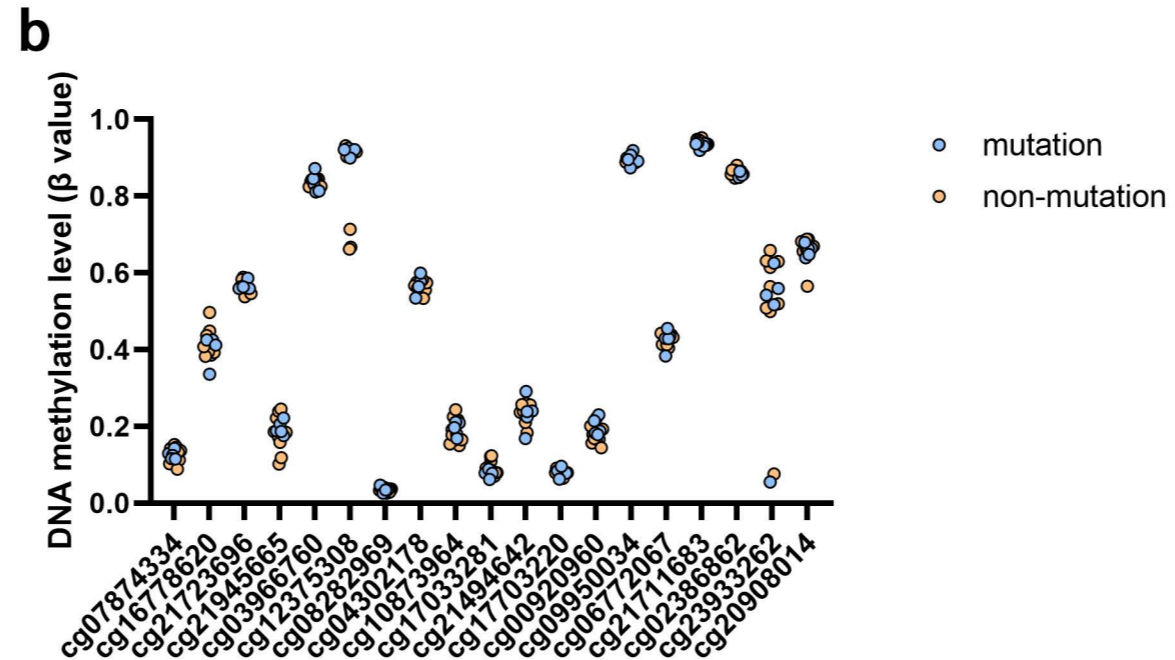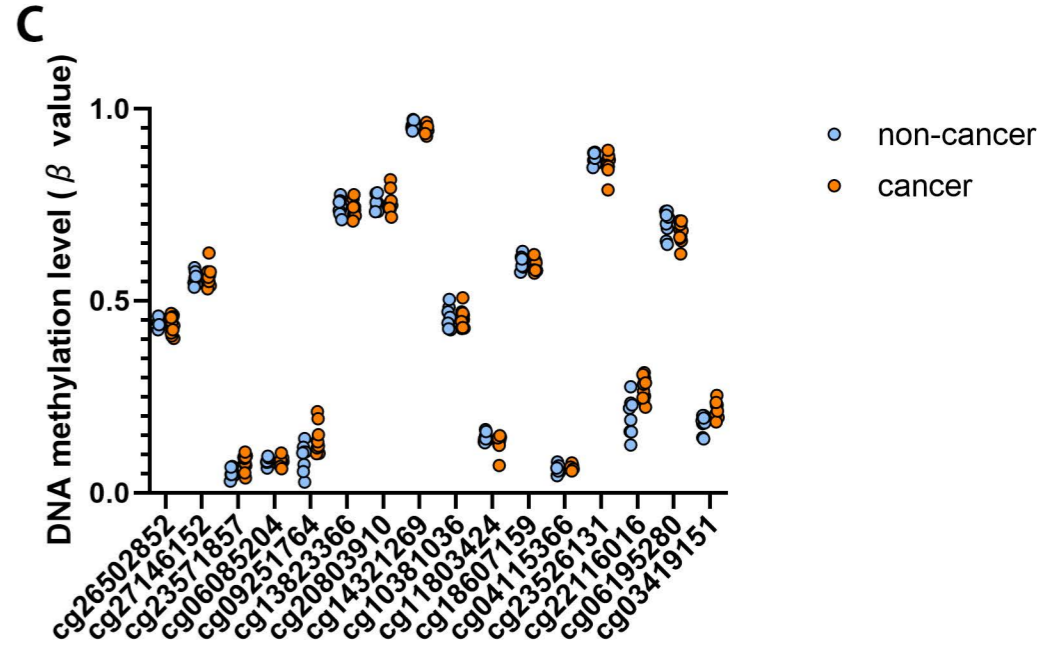

Supplement: Supplementary file 4 — Supporting Information 4 Figure S4. DNA methylation levels of XAF1 and XIAP genes. (a) DNA methylation patterns in the promoter region of the XAF1 gene. Blue: GI cancer patients carrying the XAF1 mutation (c.454+1372G>A). Orange: GI cancer patients without this mutation. (b) DNA methylation patterns in the promoter region of the XIAP gene. Blue: GI cancer patients carrying the XAF1 mutation (c.454+1372G>A). Orange: GI cancer patients without this mutation. (c) DNA methylation patterns in the promoter region of the XAF1 gene. Blue: cancer‐free individuals carrying the XAF1 mutation (c.454+1372G>A). Orange: GI cancer patients with this mutation. [file HUMU-2026-4279712-s002.pdf]

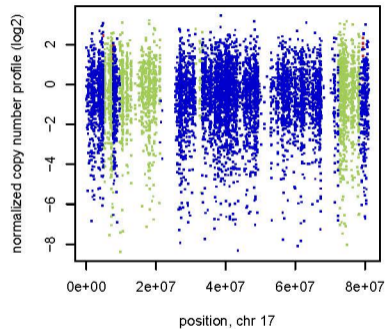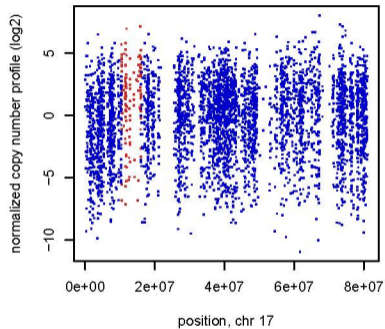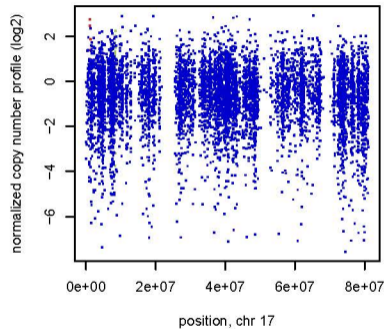

Supplement: Supplementary file 5 — Supporting Information 5 Figure S5. Copy number variations (CNVs) of Chromosome 17. The distribution of copy number variations across Chromosome 17 in three patients. Blue: decreased copy number; green: copy number neutral; red: increased copy number. [file HUMU-2026-4279712-s001.pdf]
