# Supplementary material for: Identification of a Germline XAF1 Mutation in Patients With Gastrointestinal Cancers
Source: Hum Mutat. 2026 May 4;2026:4279712. doi: 10.1155/humu/4279712 (PMC13139716; doi:10.1155/humu/4279712)
Supplement: Supplementary file 6 — Supporting Information 6 Table S1. Alternative splicing of the XAF1 transcript and isoforms of the XAF1 protein. NMD, nonsense‐mediated mRNA decay. [file HUMU-2026-4279712-s005.docx]

| **Transcript variant** | **Exons** | **mRNA length** | **CDS** | **Protein isoform** | **Protein size** | **Zinc finger domain** | **Protein molecular weight** | **Genomic change** | **Coding change** | **Protein change** |
| --- | --- | --- | --- | --- | --- | --- | --- | --- | --- | --- |
| V1 | 1, 2, 3, 4, 5, 6, 7 | 3417bp | 34-939 | Isoform 1 | 302aa | ZF1, 2, 3, 4, 5, 6, 7 | 34 KDa | NC_000017.11:g.6761973G>A | c.422-182G>A | No |
| V7 | 1(3’ extended), 2, 3, 4, 5, 6, 7 | 3626bp | 243-1148 |  |  |  |  |  |  |  |
| V2 | 1, 2, 4, 5, 6, 7 | 3360bp | 34-882 | Isoform 2 | 283aa | ZF1, 2, 4, 5, 6, 7 | 32 KDa |  | c.365-182G>A | No |
| V3 | 1, 2, 3, 4, 6, 7 | 3331bp | 101-853 | Isoform 6 | 251aa | ZF7 | 27 KDa |  | c.354+1372G>A | No |
| V4 | 1, 2, 4, 6, 7 | 3274bp | 101-796 | Isoform 7 | 232aa | ZF7 | 25 KDa |  | c.297+1372G>A | No |
| V5 | 1, 2, 5, 6, 7 | 3164bp | / | NMD | / | / | / |  | / | / |
| V6 | 1, 2, 3, 4, 4b, 5, 6, 7 | 3572bp | 577-1095 | Isoform 3 | 173aa | ZF6, 7 | 19 KDa |  | c.-35G>A | No |
| V8 | 1, 2,3 (5’ extended), 4, 5, 6, 7 | 3823bp | 620-1345 | Isoform 4 | 242aa | ZF3, 4, 5, 6, 7 | 27 KDa |  | c.242-182G>A | No |
| V9 | 1, 2, 4, 4b, 5, 6, 7 | 3512bp | 420-1034 | Isoform 5 | 205aa | ZF6, 7 | 23 KDa |  | **c.66G>A** | **p.22W>*** |
| V10 | 1, 2, 3, 4, 4b, 5, 6, 7 | 3569bp | 477-1091 |  |  |  |  |  |  |  |

Table S1 Alternative splicing of XAF1 transcript and isoforms of the XAF1 protein.

NMD, nonsense-mediated mRNA decay.

Fig S1. The Western blot of various XAF1 protein isoforms in normal esophageal and gastric tissues.


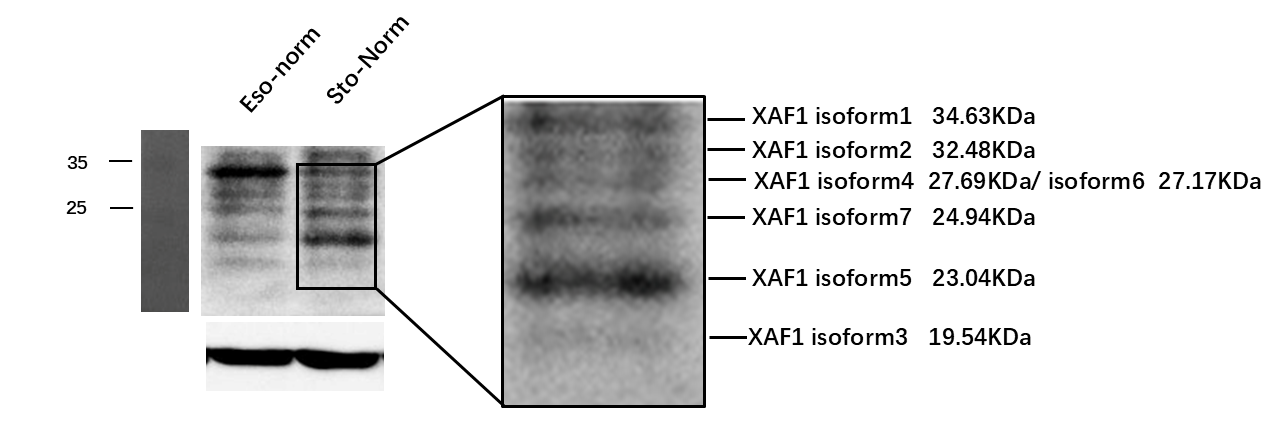


The Western blot results showed multiple band patterns for the XAF1 antibody, suggesting the expression of various XAF1 protein isoforms

Fig S2. XAF1 expression in normal gastric tissue.


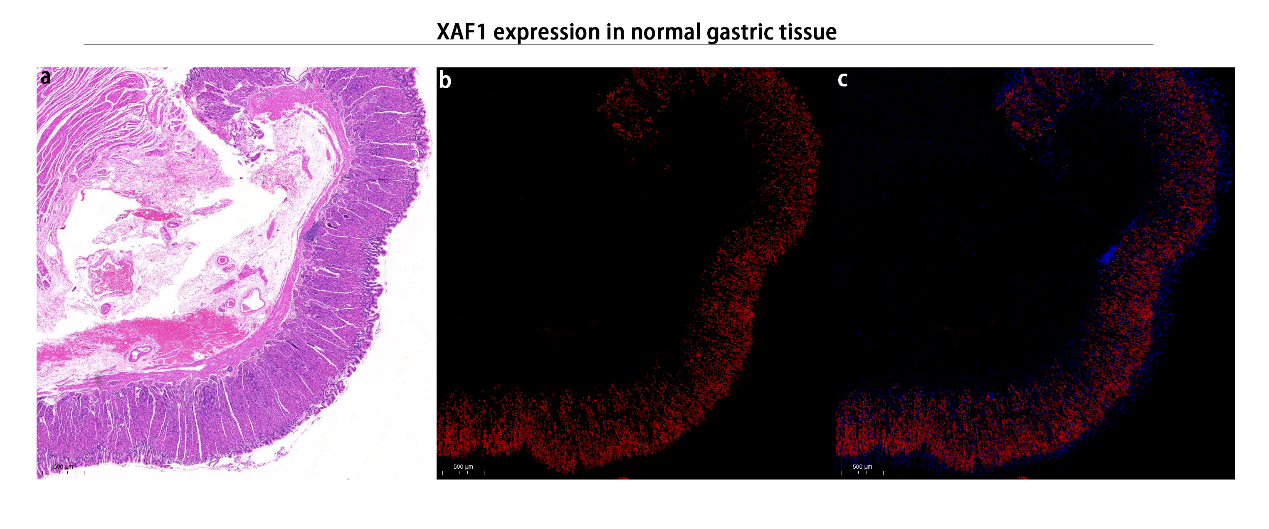


Immunofluorescence staining shows high expression of XAF1 in the normal gastric mucosal epithelium. a: HE stain; b: XAF1 expression shown as red fluorescence; c: DAPI staining shown as blue fluorescence.

Fig S3. XAF1 expression in normal esophageal tissue.


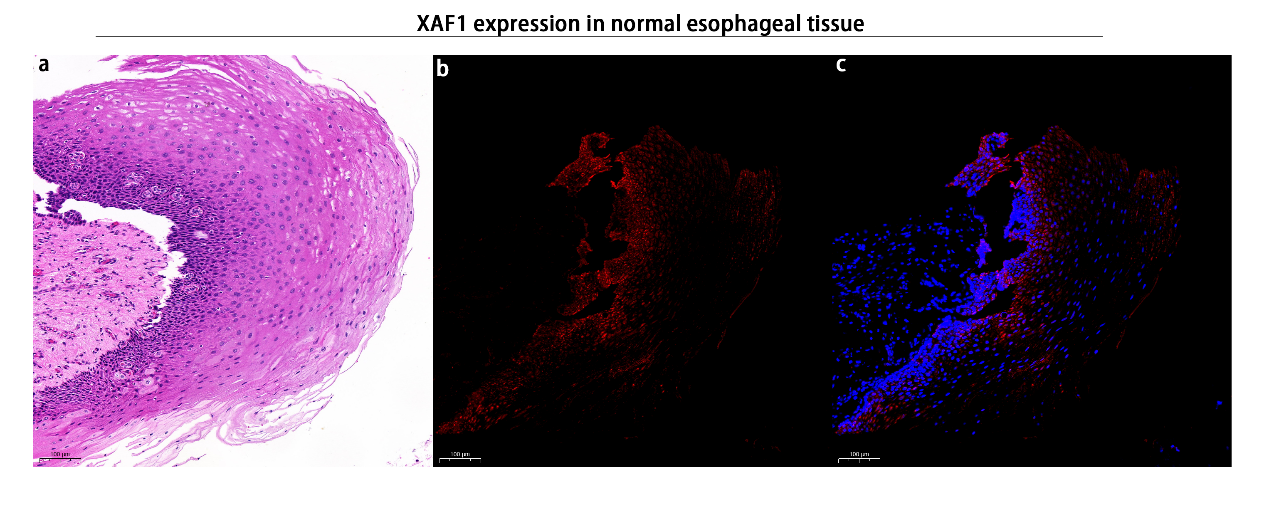


Immunofluorescence staining demonstrated high expression of XAF1 in normal esophageal tissue. a: HE stain; b: XAF1 expression shown as red fluorescence; c: DAPI staining shown as blue fluorescence.

Fig S4. DNA Methylation levels of XAF1 and XIAP genes.


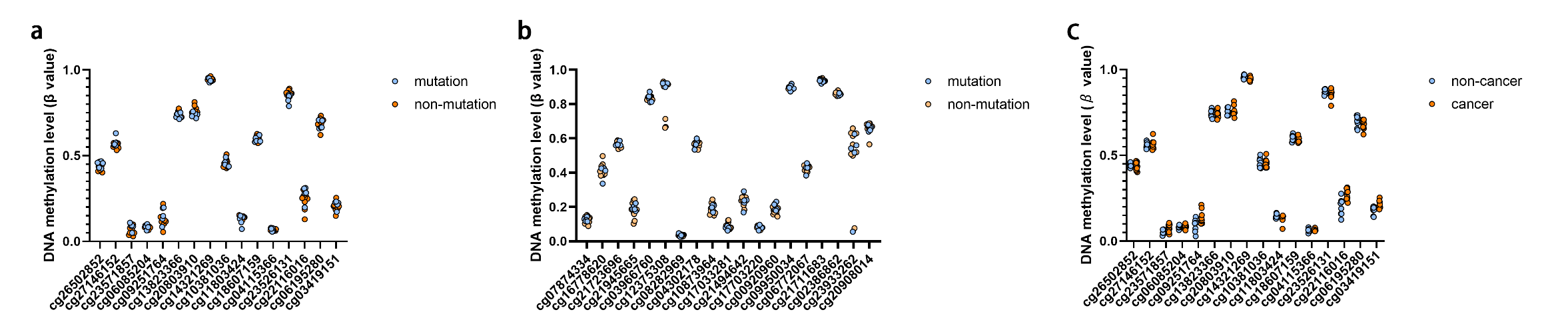


a: DNA methylation patterns in the promoter region of the *XAF1* gene. Blue: GI cancer patients carrying the *XAF1* mutation (c.454+1372G>A). Orange:GI cancer patients without this mutation. b: DNA methylation patterns in the promoter region of the *XIAP* gene. Blue: GI cancer patients carrying the *XAF1* mutation (c.454+1372G>A). Orange:GI cancer patients without this mutation. c: DNA methylation patterns in the promoter region of the *XAF1* gene. Blue: cancer-free individuals carrying the *XAF1* mutation (c.454+1372G>A). Orange: GI cancer patients with this mutation.

Fig S5. Copy number variations (CNV) of chromosome 17.


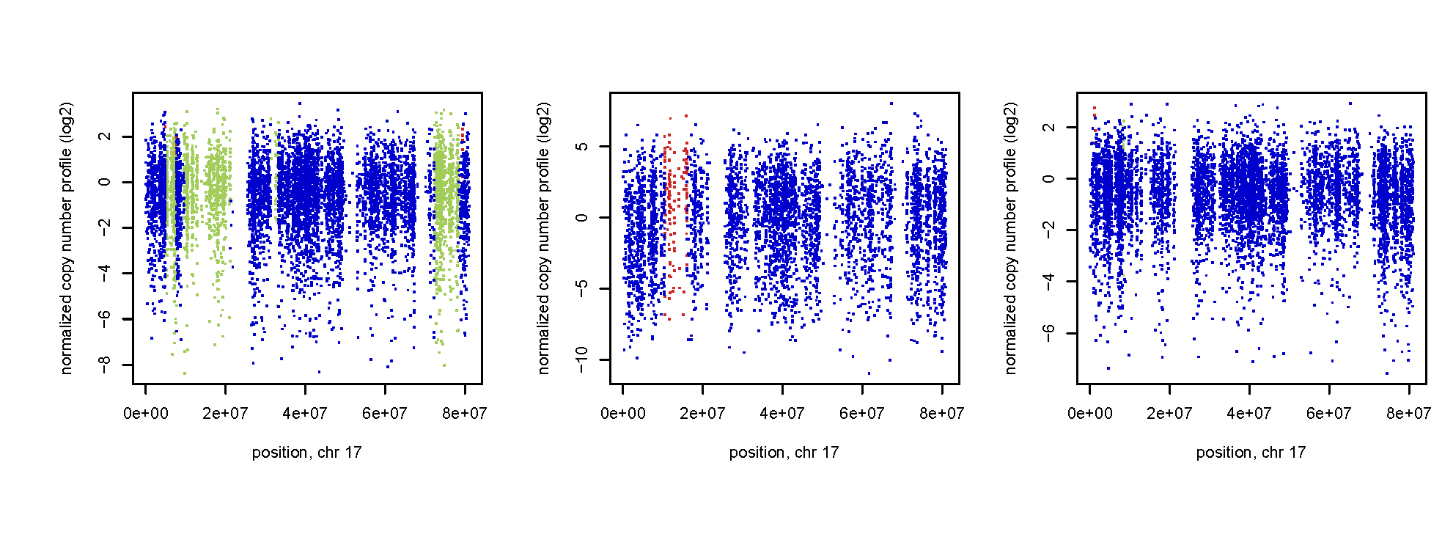


The distribution of copy number variations across chromosome 17 in three patients. Blue: decreased copy number, green: Copy number neutral, red: increased copy number.

Table S2. Transcription variants and protein isoforms of XAF1 gene.
